# Supplementary material for: Relationship of sociodemographic and lifestyle factors and diet habits with metabolic syndrome (MetS) among three ethnic groups of the Malaysian population
Source: PLoS One. 2020 Mar 19;15(3):e0224054. doi: 10.1371/journal.pone.0224054 (PMC7082049; doi:10.1371/journal.pone.0224054)
Supplement: S1 File — (DOCX) [file pone.0224054.s001.docx]

# Questionnaire

**Tabiat Permakanan dan Gaya Hidup dalam**

**Sindrom Kardiometabolik:**

**Perangkaan Program Sokongan Kesihatan Komuniti**

***Nutrition and Lifestyle Behaviours in Cardiometabolic Syndrome: Development of a Community-Specific Peer-Support Programme***

**Fasa 1 (Kulai)**

***Phase 1 (Kulai)***

| Arahan *Instructions*  Semua bahagian soal selidik ini hendaklah dilengkapkan.  *All parts of the questionnaire should be completed.*  Sila tandakan (√) atau (X) jawapan anda, melainkan jika dinyatakan sebaliknya.  *Please put a tick (√) or a cross (X) against your response, unless it is indicated otherwise.*  Ruang yang disediakan untuk anda untuk menulis pandangan anda. Sekiranya anda memerlukan lebih banyak ruang untuk menulis, sila sertakan kertas tambahan.  *Spaces are provided for you to write your views on the subject. Should you need more space to write, please feel free to attach additional sheets.*  Sila berikan pendapat yang jujur dan butir-butir yang anda mampu.  Hanya para penyelidik projek ini akan mempunyai akses kepada data anda.  Semua maklumat yang anda berikan di sini adalah sulit.  *Please provide honest opinions and as much details as you can.*  *Only the Investigators of the project will have access to the data.*  *All information you provide here will remain confidential.*  Terima kasih kerana bersetuju untuk mengambil bahagian dalam kajian ini.  Thank you for agreeing to take part in this study | CHECKLIST | | |
| --- | --- | --- | --- |
|  | - | Consent form & Screening |  |
|  | A | Personal info & sociodemography |  |
|  | B | Dietary behaviour |  |
|  | C | Physical activity (IPAQ) |  |
|  | D | Lifestyle habits |  |
|  | E | QOL |  |
|  | F | Health screening |  |
|  | G | Lab report |  |
|  | H | Weight management strategies |  |
|  | I | Food insecurity |  |
|  | J | 24-hour dietary recall |  |
|  | K | FFQ |  |
|  | L | Medical info |  |

| BORANG KEIZINAN (FASA 1) |
| --- |

Tajuk Kajian: Tabiat Pemakanan dan Gaya Hidup dalam Sindrom Kardiometabolik: Perangkaan Program Sokongan Kesihatan Komuniti

Ketua Penyelidik: Dr Amutha Ramadas

Saya bersetuju untuk mengambil bahagian dalam kajian Monash University seperti di atas. Perihal dan butiran kajian telah diterangkan kepada saya. Saya telah membaca borang penjelasan kajian yang akan saya simpan sebagai rekod.

| Saya juga bersetuju untuk: | Ya | Tidak |
| --- | --- | --- |
| *Membenarkan sampel darah diambil* |  |  |
| *Membenarkan tekanan darah, tinggi, berat, pinggang dan ukuran lemak dalam badan diambil* |  |  |
| *Ditemuramah untuk maklumat berkaitan butir-butir diri, pemakanan, gaya hidup dan kesihatan* |  |  |
| *Dihubungi semula untuk Fasa 2 dan 3* |  |  |
| *Dihubungi semula untuk kajian-kajian seterusnya* |  |  |

Saya akui bahawa penglibatan saya adalah secara sukarela, dan saya boleh memilih untuk tidak melibatkan diri ataupun boleh menarik diri dari sebarang tahap pengajian tanpa sebarang masalah.

Saya faham bahawa sebarang maklumat yang diperoleh daripada temuduga ini untuk laporan tidak mengandungi sebarang nama atau ciri-ciri peserta.

Saya faham maklumat yang saya berikan adalah peribadi dan tidak akan dibocorkan dalam laporan atau kepada pihak ketiga.

Laporan dan maklumat yang direkod dalam temuduga akan disimpan dengan selamat dan hanya akan diakses oleh pihak penyelidik kajian dan akan dimusnahkan selepas tamat kajian.

| Tandatangan |  |
| --- | --- |
| Nama Penuh |  |
| MyKad |  |
| Alamat |  |
| No Telefon |  |
| Tarikh |  |

| CONSENT FORM (PHASE 1) |
| --- |

Project: Nutrition and Lifestyle Behaviours in Cardiometabolic Syndrome: Development of a Community-Specific Peer-Support Programme

Chief Investigator: Dr Amutha Ramadas

I agree to take part in the Monash University research project specified above. I have had the project explained to me, and I have read the Explanatory Statement, which I keep for my records.

| I consent to the following: | Yes | No |
| --- | --- | --- |
| *Taking my blood sample* |  |  |
| *Taking my blood pressure, height, weight, waist and body fat measurements* |  |  |
| *Asking questions regarding my basic details, nutrition, lifestyle and quality of life* |  |  |
| *To be recontacted for Phase 2 and Phase 3* |  |  |
| *To be recontacted for future research* |  |  |

I understand that my participation is voluntary, that I can choose not to participate in part or all of the project, and that I can withdraw at any stage of the project without being penalised or disadvantaged in any way.

I understand that any data from the interview for use in reports will not, under any circumstances, contain names or identifying characteristics.

I understand that any information I provide is confidential, and that no information that could lead to the identification of any individual will be disclosed in any reports on the project, or to any other party.

I understand that reports based on the interview(s) will be kept in a secure storage and accessible only to the research team. I also understand that the collected data will be destroyed after the stipulated period.

| Signature |  |
| --- | --- |
| Full name |  |
| MyKad |  |
| Address |  |
| Tel no. |  |
| Date |  |

SCREENING QUESTIONNAIRE

Note: To be administered by the study researcher at Registration Counter

Tick (√) all characteristics that describe the individual

| Inclusion criteria | | |
| --- | --- | --- |
| 1 | Age ≥ 18 years at the time of the study |  |
| 2 | Malaysian citizen |  |

| Exclusion criteria | | |
| --- | --- | --- |
| 1 | Pregnant women |  |
| 2 | Psychiatric illness |  |
| 3 | Subject with malignancy |  |
| 4 | Drug/alcohol abuser |  |
| 5 | End stage renal failure |  |
| 6 | Chronic liver diseases including hepatoma |  |
| 7 | Immuno-compromised subject such as HIV |  |
| 8 | Cognitive impairment |  |
| 9 | Speech difficulty |  |
| 10 | Hearing difficulty |  |
| 11 | Non-ambulatory |  |

| A. MAKLUMAT SOSIO-DEMOGRAFI / *SOCIO-DEMOGRAPHIC INFORMATION* | | | | | | |
| --- | --- | --- | --- | --- | --- | --- |
|  | | | | | | |
| 1. | Umur:  *Age:* | ____________tahun / *years* |  | 7. | Pendapatan individu (RM):  *Personal income (RM):* | ____________________ |
| 2. | Jantina:  *Sex:* | (1) Lelaki / *Male*  (2) Perempuan / *Female* |  |  |  |  |
| 3. | Status perkahwinan:  *Marital status:* | (1) Bujang / *Single*  (2) Berkahwin / *Married*  (3) Bersekedudukan / *Living with partner*  (4) Janda/duda / *Widowed*  (5) Bercerai / *Divorced*  (6) Berpisah / *Separated* |  | 8. | Pendapatan isirumah (RM):  *Household income (RM):* | ______________________ |
| 4. | Etnik:  *Ethnicity:* | (1) Melayu / *Malay*  (2) Cina / *Chinese*  (3) India / *India*  (4) Lain-lain/ *Others* _________________ |  | 9. | Bilangan orang dalam isirumah:  *Number of people in the household:* | _________________ |
| 5. | Pendidikan:  *Education:* | (1) Rendah / *Primary*  (2) Menengah rendah /*Lower secondary*  (3) Menengah tinggi/ *Higher secondary*  (4) Tertiari/tinggi/ *Tertiary* |  | 10. | Bilangan tahun bermastautin:  *Number of years of residence:* | _______________tahun / *years* |
| 6. | Pekerjaan:  *Occupation:* | (1) Pelajar / *Student*  (2) Bekerja / *Employed*  (3) Tidak bekerja / *Unemployed*  (4) Bersara / *Retired* |  |  |  |  |

| B. TABIAT PEMAKANAN / *DIETARY BEHAVIOUR* | | | |
| --- | --- | --- | --- |
|  | | | |
| 1. Adakah anda seorang vegetarian?   *Are you a vegetarian?* | | (1) Ya / *Yes*  Jenis / *Type: ____________________________*  (2) Tidak / *No* | |
| 1. Berapa cepat anda habiskan makanan anda?   *How fast do you finish your meals?* | | (1) 30 minit atau lebih / 3*0 minutes or more*  (2) 10-29 minit / 1*0-29 minutes*  (3) kurang dari 10 minit / *less than 10 minutes* | |
| 1. Berapa kerap anda makan selepas 10 malam?   *How frequent do you eat after 10pm?* | | (1) Setiap hari / *Every day*  (2) 4 – 6 kali seminggu / 4*-6 times a week*  (3) 1 – 3 kali seminggu / *1-3 times a week*  (4) Kurang dari sekali seminggu / *Less than once a week*  (5) Tidak pernah atau jarang / *Never or rare* | |
| 1. Berapa kerap anda mengelakkan makan sarapan?   *How frequent do you skip your breakfast?* | | (1) Setiap hari / *Every day*  (2) 4 – 6 kali seminggu / 4*-6 times a week*  (3) 1 – 3 kali seminggu / *1-3 times a week*  (4) Kurang dari sekali seminggu / *Less than once a week*  (5) Tidak pernah atau jarang / *Never or rare* | |
| 1. Berapa kerap anda makan di luar?   *How frequent do you dine out?* | | (1) Setiap hari / *Every day*  (2) 4 – 6 kali seminggu / 4*-6 times a week*  (3) 1 – 3 kali seminggu / *1-3 times a week*  (4) Kurang dari sekali seminggu / *Less than once a week*  (5) Tidak pernah atau jarang / *Never or rare* | |
| 1. Adakah anda aktif bersukan atau bergim?   *Are you active in sports or gym?* | | (1) Ya / *Yes*  (2) Tidak / *No* | |
| 1. Adakah anda mengambil sebarang suplemen makanan?   *Do you consume any nutritional supplement?* | | (1) Ya  (2) Tidak *(Skip to Section C / Sila ke Bhg. C)* | |
| 1. Adakah anda mendapat nasihat dari doktor / pakar pemakanan sebelum mengambil suplemen?   *Did you seek advice from doctor or nutritionist before consuming supplements?* | | (1) Ya / *Yes*  (2) Tidak / *No* | |
| 1. Adakah anda lebih memilih atau mempercayai suplemen berbanding ubat-ubatan moden / diberikan oleh doktor?   *Do you choose/trust supplements more than modern medicine / drugs given by doctor?* | | (1) Ya / *Yes*  (2) Tidak / *No* | |
| 1. Mengapakah anda mengambil supplemen pemakanan?   *Why do you take supplements?* | (1) Merawat penyakit:*Treat disease:* ______________________________  (2) Menambahbaik diet seharian  *Improve my daily diet*  (3) Mengikut nasihat rakan / ahli keluarga  *Friends’ or family members’ advice*  (4) Saranan doctor  *Doctor’s advice*  (5) Lain-lain / *others*: ________________ | |  |
| \|  \| \| \| \| \|  \| \| --- \| --- \| --- \| --- \| --- \| --- \| \| C. AKTIVITI FIZIKAL / *PHYSICAL ACTIVITY* \| \| \| \| \|  \| \|  \|  \|  \| \| \|  \| \| Soalan-soalan berikut akan menyoal anda tentang jumlah masa yang anda gunakan untuk berada dalam keadaan aktif secara fizikal dalam tempoh 7 hari yang lepas ini.  The questions will ask you about the time you spent being physically active in the last 7 days. \| \| \| \| \| \| \|  \| \| \| \| \|  \| \| Fikirkan tentang semua aktiviti fizikal berat yang anda telah lakukan dalam tempoh 7 hari yang lepas ini. Aktiviti fizikal berat adalah aktiviti yang menggunakan daya tenaga fizikal yang kuat dan membuat anda bernafas jauh lebih kuat daripada biasa. Fikirkan hanya tentang aktiviti-aktiviti fizikal yang anda telah lakukan selama sekurang-kurangnya 10 minit pada sesuatu masa.  *Think about all the vigorous activities that you did in the last 7 days. Vigorous physical activities refer to activities that take hard physical effort and make you breathe much harder than normal. Think only about those physical activities that you did for at least 10 minutes at a time.* \| \| \| \| \| \| \|  \| \| \| \| \|  \| \| 1. \| Dalam tempoh 7 hari yang lepas ini, berapa harikah anda telah melakukan aktiviti fizikal berat, contohnya mengangkat barang berat, mencangkul, senaman aerobik atau berbasikal laju?  *During the last 7 days, how many days did you do vigorous physical activities such as heavy lifting, digging, aerobics, or fast bicycling, or outdoor games (in days/week)?* \| \| ________hari/seminggu  *days/week*  (0) Tiada *(sila ke S3)*  *None (skip to Q3)* \| \| *METs Factor- 8.0 \| \|  \| \| \| \| \| \| 2. \| Berapakah masa yang anda biasa gunakan untuk melakukan aktiviti fizikal berat pada salah satu daripada hari berkenaan?  *How much time did you spend doing vigorous physical activities on one of those days (in minutes/day)?* \| \| ________minit/sehari  *minutes/day*  (0) Tidak tahu / tidak pasti  *Don’t know / not sure* \| \| \|  \|  \| \|  \| \|  \| \| Think about all the moderate activities that you did in the last 7 days. Moderate activities refer to activities that take moderate physical effort and make you breathe somewhat harder than normal. Think only about those physical activities that you did for at least 10 minutes at a time.  *Fikirkan tentang semua aktiviti fizikal sederhana yang anda telah lakukan dalam tempoh 7 hari yang lepas ini. Aktiviti fizikal sederhana adalah aktiviti yang menggunakan daya tenaga fizikal yang sederhana dan membuatkan anda bernafas agak lebih kuat daripada biasa. Fikirkan hanya tentang aktiviti-aktiviti fizikal yang anda telah lakukan selama sekurang-kurangnya 10 minit pada sesuatu masa.* \| \| \| \| \| \| \|  \| \| \| \| \|  \| \| 3. \| Dalam tempoh 7 hari yang lepas ini, berapa harikah anda telah melakukan aktiviti fizikal sederhana, contohnya mengangkat muatan ringan, mengelap lantai, berbasikal pada kelajuan biasa, atau bermain badminton beregu? Ini tidak termasuk berjalan kaki.  *During the last 7 days, on how many days did you do moderate physical activities like carrying light loads, bicycling at a regular pace, or doubles tennis? (Do not include walking).* \| \| ________hari/seminggu  *days/week*  (0) Tiada *(sila ke S5)*  *None (skip to Q5)* \| \| *METs Factor- 4.0 \| \|  \| \| \| \| \| \| 4. \| Berapakah masa yang anda biasa gunakan untuk melakukan aktiviti fizikal sederhana pada salah satu daripada hari berkenaan?  *How much time did you spend doing moderate physical activities on one of those days (in minutes/day)?* \| \| ________minit/sehari  *minutes/day*  (0) Tidak tahu / tidak pasti  *Don’t know / not sure* \| \| \|  \|  \| \|  \| \|  \| \| Think about the time you spent walking in the last 7 days. This includes at work and at home, walking to travel from place to place, and any other walking that you might do solely for recreation, sport, exercise, or leisure.  *Fikirkan tentang masa yang anda telah gunakan untuk berjalan kaki dalam tempoh 7 hari yang lepas ini. Masa ini merangkumi berjalan kaki di tempat kerja dan di rumah, berjalan kaki dari satu tempat ke tempat yang lain, dan berjalan kaki semata-mata untuk rekreasi, bersukan, bersenam atau pada masa lapang.* \| \| \| \| \| \| \|  \|  \| \|  \| \|  \| \| 5. \| Dalam tempoh 7 hari yang lepas ini, berapa harikah anda telah berjalan kaki selama sekurang-kurangnya 10 minit pada sesuatu masa?  *During the last 7 days, on how many days did you walk for at least 10 minutes at a time?* \| \| ________hari/seminggu  *days/week*  (0) Tiada *(sila ke S7)*  *None (skip to Q7)* \| \| *METs Factor- 3.3 \| \|  \| \| \| \| \| \| 6. \| Berapakah masa yang anda biasa gunakan untuk berjalan kaki pada salah satu daripada hari berkenaan?  *How much time did you usually spend walking on one of those days (in minutes/day)?* \| \| ________minit/sehari  *minutes/day*  (0) Tidak tahu / tidak pasti  *Don’t know / not sure* \| \| \|  \|  \| \|  \| \|  \| \| The last question is about the time you spent sitting on weekdays during the last 7 days. Include time spent at work, at home, while doing course work and during leisure time. This may include time spent sitting at a desk, visiting friends, reading, or sitting or lying down to watch television.  *Soalan terakhir ini adalah berkaitan masa yang anda telah gunakan untuk duduk pada hari-hari bekerja dalam tempoh 7 hari yang lepas ini. Masukkan masa yang di habiskan duduk di tempat kerja, di rumah, sewaktu belajar dan di masa lapang. Masa ini juga merangkumi waktu yang di habiskan duduk di meja, menziarahi kawan-kawan, membaca, atau duduk atau baring sambil menonton televisyen.* \| \| \| \| \| \| \|  \| \| \| \| \|  \| \| 7. \| Dalam tempoh 7 hari yang lepas ini, berapakah masa yang anda telah gunakan untuk duduk pada sesuatu hari bekerja.  *During the last 7 days, how much time did you spend sitting on a week day (in minutes/day)?* \| \| ________minit/sehari  *minutes/day*  (0) Tidak tahu / tidak pasti  *Don’t know / not sure* \| \|  \| \| METs \| \| \| \| \|  \| \|  \|  \| \|  \| \|  \| \|  \| Tahap aktiviti fizikal  *Level of physical activity* \| \| \| aktif (>3000)  *active*  sederhana (700-2900)  *moderately active*  tidak aktif (<600)  *not active* \| \| | | |  |

| D. GAYA HIDUP / *LIFESTYLE HABITS* | | |
| --- | --- | --- |
|  | | |
| 1. Pernahkah anda merokok?   *Do/did you smoke?* | (1) Tidak merokok / *non-smoker* *(Sila ke S4/skip to Q4)*  (2) Pernah merokok / *past-smoker*  (3) Masih merokok / *current smoker* | |
| 1. Berapa lama anda telah merokok?   *How long have you been smoking?* | ________________________ tahun / *years* | |
| 1. Berapa batang rokok anda hisap sehari?   *How many cigarettes do/did you smoke per day?* | ________________________ batang / *sticks* | |
| 1. Pernahkah anda minum arak?   *Do/did you drink alcohol?* | (1) Tidak minum / *non-drinker* *(sila ke S6 /skip to Q6)*  (2) Pernah minum / *past-drinker*  (3) Masih minum / *current drinker* | |
| 1. Berapa lama anda minum arak?   *How long have you been consuming alcohol?* | ______________________ tahun / *years* | |
| 1. Berapa lama anda tidur dalam sehari?   *How many hours do you sleep in a day?* | (1) kurang dari 6 jam / *less than 6 hours*  (2) 6-8 jam / 6*-8 hours*  (3) lebih dari 8 jam / *more than 8 hours* | |
|  | |  |

| E. KUALITI HIDUP / *QUALITY OF LIFE* | | | | | | |
| --- | --- | --- | --- | --- | --- | --- |
| Soalan-soalan berikut bertanyakan pandangan anda tentang kualiti hidup, kesihatan atau aspek-aspek kehidupan yang lain. Pilih jawapan yang anda rasa paling bersesuaian. Sila ambil perhatian terhadap standad, harapan, keseronokan dan kebimbangan anda. Fikirkan tentang kehidupan anda dalam tempoh empat minggu lepas.  *The following questions ask how you feel about your quality of life, health, or other areas of your life. I will read out each question to you, along with the response options. Please choose the answer that appears most appropriate. If you are unsure about which response to give to a question, the first response you think of is often the best one. Please keep in mind your standards, hopes, pleasures and concerns. We ask that you think about your life in the last four weeks.* | | | | | | |
|  | Sangat tidak baik  *Very poor* | Tidak baik  *Poor* | Sederhana  *Neither poor nor good* | | Baik  *Good* | Sangat baik  *Very good* |
| Bagaimanakah anda menilai kualiti kehidupan anda?  *How would you rate your quality of life?* | 1 | 2 | 3 | | 4 | 5 |
|  | Sangat tidak berpuas hati  *Very dissatisfied* | Tidak berpuas hati  *Dissatisfied* | Sederhana  *Neither satisfied nor dissatisfied* | | Berpuas hati  *Satisfied* | Sangat berpuas hati  *Very satisfied* |
| Setakat manakah anda berpuas hati dengan kesihatan anda?  *How satisfied are you with your health?* | 1 | 2 | 3 | | 4 | 5 |
|  |  |  |  |  |  |  |
| Soalan-soalan berikutnya bertanyakan setakat mana anda telah mengalami sesuatu perkara dalam 4 minggu yang lepas.  *The following questions ask about how much you have experienced certain things in the last four weeks.* | | | | | | |
|  | Tiada langsung  *Not at all* | Sedikit sahaja  *A little* | Sederhana  *A moderate amount* | | Sangat banyak  *Very much* | Teramat  *An extreme amount* |
| Setakat manakah anda berasa kesakitan (fizikal) menghalang anda dari melakukan apa yang anda perlu lakukan?  *To what extent do you feel that physical pain prevents you from doing what you need to do?* | 5 | 4 | 3 | | 2 | 1 |
|  | Tiada langsung  *Not at all* | Sedikit sahaja  *A little* | Sederhana  *A moderate amount* | | Sangat banyak  *Very much* | Teramat  *An extreme amount* |
| Berapa banyakkah rawatan perubatan yang anda perlu untuk berfungsi dalam kehidupan harian anda?  *How much do you need any medical treatment to function in your daily life?* | 5 | 4 | 3 | | 2 | 1 |
| Berapa banyakkah anda menikmati keseronokan dalam hidup anda?  *How much do you enjoy life?* | 1 | 2 | 3 | | 4 | 5 |
| Setakat manakah anda rasa hidup anda bermakna?  *To what extent do you feel your life to be meaningful?* | 1 | 2 | 3 | | 4 | 5 |
| Berapa baikkah anda dapat memberi tumpuan?  *How well are you able to concentrate?* | 1 | 2 | 3 | | 4 | 5 |
| Berapa selamatkah anda rasa dalam kehidupan seharian anda?  *How safe do you feel in your daily life?* | 1 | 2 | 3 | | 4 | 5 |
| Berapa sihatkah persekitaran fizikal anda?  *How healthy is your physical environment?* | 1 | 2 | 3 | | 4 | 5 |
|  |  |  |  |  |  |  |
| Soalan-soalan berikut bertanyakan bagaimana sempurnanya anda mengalami atau berupaya melakukan sesuatu perkara dalam 4 minggu yang lepas.  *The following questions ask about how completely you experience or were able to do certain things in the last four weeks.* | | | | | | |
|  | Tiada langsung  *Not at all* | Sedikit sahaja  *A little* | Sederhana  *Moderately* | | Kebanyakkannya  *Mostly* | Sepenuhnya  *Completely* |
| Adakah anda mempunyai cukup tenaga untuk kehidupan harian anda?  *Do you have enough energy for everyday life?* | 1 | 2 | 3 | | 4 | 5 |
| Adakah anda dapat menerima rupa dan betuk tubuh anda?  *Are you able to accept your bodily appearance?* | 1 | 2 | 3 | | 4 | 5 |
| Adakah anda mempunyai wang yang cukup untuk memenuhi keperluan anda?  *Have you enough money to meet your needs?* | 1 | 2 | 3 | | 4 | 5 |
| Setakat manakah kemudahan bagi anda untuk mendapatkan maklumat yang diperlukan dalam kehidupan harian?  *How available to you is the information that you need in your day-to-day life?* | 1 | 2 | 3 | | 4 | 5 |
| Setakat manakah anda mendapat peluang untuk aktiviti fizikal?  *To what extent do you have the opportunity for leisure activities?* | 1 | 2 | 3 | | 4 | 5 |
| Sebaik manakah keupayaan anda bergerak dari satu tempat ke satu tempat yang lain?  *How well are you able to get around?* | 1 | 2 | 3 | | 4 | 5 |

|  |  |  |  | |  |  |
| --- | --- | --- | --- | --- | --- | --- |
|  | Sangat tidak berpuas hati  *Very dissatisified* | Tidak berpuas hati  *Dissatisfied* | Sederhana  *Neither satisfied nor dissatisfied* | | Berpuas hati  *Satisfied* | Sangat berpuas hati  *Very satisfied* |
| Adakah anda berpuas hati dengan tidur anda?  *How satisfied are you with your sleep?* | 1 | 2 | 3 | | 4 | 5 |
| Adakah anda berpuas hati dengan keupayaan anda melaksanakan aktiviti kehidupan harian anda?  *How satisfied are you with your ability to perform your daily living activities?* | 1 | 2 | 3 | | 4 | 5 |
| Adakah anda berpuas hati dengan keupayaan anda bekerja?  *How satisfied are you with your capacity for work?* | 1 | 2 | 3 | | 4 | 5 |
| Adakah anda berpuas hati dengan diri anda?  *How satisfied are you with yourself?* | 1 | 2 | 3 | | 4 | 5 |
| Adakah anda berpuas hati dengan perhubungan peribadi anda?  *How satisfied are you with your personal relationships?* | 1 | 2 | 3 | | 4 | 5 |
| Adakah anda berpuas hati dengan kehidupan seks anda?  *How satisfied are you with your sex life?* | 1 | 2 | 3 | | 4 | 5 |
| Adakah anda berpuas hati dengan sokongan yang anda dapati dari kawan-kawan anda?  *How satisfied are you with the support you get from your friends?* | 1 | 2 | 3 | | 4 | 5 |
| Adakah anda berpuas hati dengan keadaan tempat tinggal anda?  *How satisfied are you with the conditions of your living place?* | 1 | 2 | 3 | | 4 | 5 |
| Adakah anda berpuas hati dengan kemudahan mendapatkan perkhidmatan kesihatan?  *How satisfied are you with your access to health services?* | 1 | 2 | 3 | | 4 | 5 |
| Adakah anda berpuas hati dengan pengangkutan anda?  *How satisfied are you with your transport?* | 1 | 2 | 3 | | 4 | 5 |
|  |  |  |  |  |  |  |

| Soalan berikut merujuk kepada kekerapan anda merasa atau mengalami sesuatu emosi sepanjang 4 minggu yang lepas.  *The following question refers to how often you have felt or experienced certain things in the last four weeks.* | | | | | |
| --- | --- | --- | --- | --- | --- |
|  | Tidak pernah  *Never* | Jarang-jarang  *Seldom* | Kerap  *Quite often* | Sangat kerap  *Very often* | Sentiasa  *Always* |
| Berapa kerapkah anda mempunyai perasaan-perasaan negative, seperti susah hati, kecewa, kegelisahan atau kemurungan?  *How often do you have negative feelings such as blue mood, despair, anxiety, depression?* | 5 | 4 | 3 | 2 | 1 |

| F. SARINGAN KESIHATAN / *HEALTH SCREENING* | | | |
| --- | --- | --- | --- |
|  |  |  |  |
|  | Parameter Kesihatan / *Health parameter* | Bacaan / *Reading* | |
| 1. | Tekanan darah sistolik (mmHg)  *Systolic blood pressure (mmHg)* |  | |
| 2. | Tekanan darah diastolik (mmHg)  *Diastolic blood pressure (mmHg)* |  | |
| 3. | Tahap gula dalam darah (puasa) (mmol/L)  *Fasting blood glucose (mmol/L)* |  | |
| 4. | Tinggi (m)  *Height (m)* |  | |
| 5. | Berat (kg)  *Weight (kg)* |  | |
| 6. | BMI (kg/m^2^)  *BMI (kg/m^2^)* |  | |
| 7. | Ukurlilit pinggang (cm)  *Waist circumference (cm)* |  | |
| 8. | Peratusan lemak badan (%)  *Body fat percentage (%)* |  | |

| G. LAPORAN MAKMAL PERUBATAN | | | | | |
| --- | --- | --- | --- | --- | --- |
|  |  |  | |  |  |
|  | Puasa (8-12 jam)  *Fasting (8-12 hours)* | Ya / *Yes*  Tidak / *No* | | | |
|  | Tiub Sampel Darah  *Blood sample tube* | Tiub Serum (Kuning) / *Serum tube (yellow)*  Tiub EDTA (Ungu) / *EDTA tube (purple)* | | | |
| 1. | Tahap serum glukosa (berpuasa) (mmol/L)  *Fasting serum glucose level (mmol/L)* |  |  |  | |
| 2. | Index HbA1c (mmol/mol)  *HbA1c index(mmol/mol)* |  |  |  | |
| 3. | Peratusan HbA1c (%)  *HbA1c percentage (%)* |  |  |  | |
| 3. | Tahap serum trigliserida (mmol/L)  *Serum triglyceride (mmol/L)* |  |  |  | |
| 4. | Tahap serum kolesterol HDL (mmol/L)  *Serum HDL cholesterol (mmol/L)* |  |  |  | |

| H. STRATEGI PENGURUSAN BERAT BADAN / *STRATEGIES FOR WEIGHT MANAGEMENT* | | | | | | | | | | | | |
| --- | --- | --- | --- | --- | --- | --- | --- | --- | --- | --- | --- | --- |
|  |  | |  |  |  | |  |  | |  | | |
| 1. | Pernahkah anda cuba mengurangkan berat badan?  *Have you ever tried to lose weight?* | | Ya / *Yes*  Tidak / *No* | | | | | | | | | |
| 2. | Pernahkah anda berjaya kurangkan sebarang berat badan?  *Have you ever lost any weight intentionally?* | | Ya / *Yes*  Tidak / *No* | | | | | | | | | |
| 3. | Jika ya, berapa kg berat anda telah kurangkan?  *If yes, how many kg have you lost?* | | ___________________________________kg | | | | | | | | | |
|  |  | |  | | | | | | | | | |
| 4. | Berapa lamakah anda telah cuba untuk kurangkan berat badan?  *How long have you been trying to lose weight?* | | ___________________________________ minggu / bulan  *weeks / months* | | | | | | | | | |
|  |  | |  | | | | | | | | | |
| Dalam tempoh masa satu bulan yang lalu, berapa kerap anda menggunakan strategi berikut untuk mengawal berat badan?  *Over the last 30 days, how often have you used the following strategies to manage your weight?* | | | | | | | | | | | | |
|  |  | Tidak Pernah  Never or hardly ever  (1) | | | | Jarang  *Some of the time*  (2) | | | Kadangkala  *About half of the time*  (3) | | Kerap  *Much of the time*  (4) | Selalu/ Hampir setiap masa  *Always or almost always*  (5) |
| SUBSKALA 1 – Jumlah Tenaga  *SUBSCALE 1 : Energy intake* | | | | | | | | | | | | |
| 1. | Elak /mengurangkan pengambilan gula atau makanan ringan  *Cut out/reduced sweets or junk food* |  | | | |  | | |  | |  |  |
| 2. | Elak / mengurangkan makan snek pada lewat malam  *Cut out/reduced late night snacking* |  | | | |  | | |  | |  |  |
| 3. | Elak / mengurangkan snek di antara makan sarapan / tengah hari / malam  *Cut out/reduced between meal snacks* |  | | | |  | | |  | |  |  |
| 4. | Mengurangkan pengambilan pencuci mulut  *Decreased frequency or portion sizes of desserts* |  | | | |  | | |  | |  |  |
| 5. | Mengurangkan kalori dalam diet  *Reduced my calorie intake* |  | | | |  | | |  | |  |  |
| 6. | Menyingkirkan makanan berkalori tinggi dari rumah/pejabat/bilik  *Removed high calorie foods from my home, office or room* |  | | | |  | | |  | |  |  |
| 7. | Mengurangkan pengambilan makanan berlemak  *Ate less fat* |  | | | |  | | |  | |  |  |
| 8. | Makan lebih banyak buah-buahan dan sayur-sayuran  *Increased fruits and vegetables* |  | | | |  | | |  | |  |  |
| SUBSKALA 2 – Penggunaan Tenaga  *SUBSCALE 2: Energy expenditure* | | | | | | | | | | | | |
| 9. | Bersenam untuk tempoh masa 30 minit atau lebih  Exercised for period of 30 minutes or more |  | | | |  | | |  | |  |  |
| 10. | Bersenam di gim atau menyertai apa-apa kelas senaman  Exercised at a gym or participated in an exercise class |  | | | |  | | |  | |  |  |
| 11. | Mengubah gaya hidup supaya dapat membuat lebih banyak aktiviti fizikal  *Altered my daily routine to get more lifestyle physical activity* |  | | | |  | | |  | |  |  |
| SUBSKALA 3 – Pemantuan  *SUBSCALE 3: Self-monitoring* | | | | | | | | | | | | |
| 12. | Merekod berat badan saya  *Recorded or graphed my weight* |  | | | |  | | |  | |  |  |
| 13. | Merekod aktiviti fizikal harian  *Recorded or graphed my physical activity* |  | | | |  | | |  | |  |  |
| 14. | Merekod jenis and kuantiti makanan yang saya makan  *Recorded or wrote down the type and quantity of food eaten* |  | | | |  | | |  | |  |  |
| 15. | Menimbang berat badan saya setiap hari atau dengan kerap  *Weighed myself regularly or daily* |  | | | |  | | |  | |  |  |
| SUBSKALA 4 – Kawalan  *SUBSCALE 4: Self-regulation* | | | | | | | | | | | | |
| 16. | Saya akan meninggalkan baki makanan di pinggan saya jika dihidang dengan terlalu banyak makanan  *If I was served too much, I left food on my plate* |  | | | |  | | |  | |  |  |
| 17. | Mengubah cara memasak  *Changed food preparation techniques* |  | | | |  | | |  | |  |  |
| 18. | Mengurangkan kuantiti pengambilan makanan  *Reduced portion sizes* |  | | | |  | | |  | |  |  |
| 19. | Merancang jenis dan kuantiti makanan dan snek yang saya makan  *Decided ahead of time what I would eat for meals and snacks* |  | | | |  | | |  | |  |  |
| 20. | Menyediakan snek sihat dan dalam kuantiti yang sesuai dengan diri saya  *Kept healthy ready-to-eat or portion controlled snacks for myself* |  | | | |  | | |  | |  |  |

|  | | | | |
| --- | --- | --- | --- | --- |
| I. KEMANDIRIAN DIET / *FOOD INSECURITY* | | | | |
|  | | | | |
| Item | | Tidak pernah  *Never* | Kadang kala  *Sometimes* | Selalu  *Often* |
| 1. | Saya bimbang sama ada makanan saya akan habis sebelum saya mendapatkan wang untuk membeli lagi  *I worry whether my food will run out before I get money to buy more* |  |  |  |
| 2. | Makanan yang saya beli tidak cukup dan saya tidak mempunyai wang untuk membeli lagi  *The food I bought just didn’t last and I didn’t have money to get more* |  |  |  |
| 3. | Saya kehabisan makanan yang diperlukan untuk memasak dan saya tidak mempunyai wang to membeli makanan lagi  *I worry about where the next day’s food is going to come from* |  |  |  |
| 4. | Saya bimbang bagaimana saya akan menyediakan makanan untuk esok hari  *I can’t afford to eat the way I should* |  |  |  |
| 5. | Saya tidak mempunyai wang untuk makan seperti yang sepatutnya  *I can’t afford to eat properly* |  |  |  |
| 6. | Saya tidak mampu untuk makan dengan betul  *I am often hungry, but I don’t eat because I can’t afford enough food* |  |  |  |
| 7. | Saya selalu lapar, tetapi saya tidak makan kerana tidak mampu membeli makanan yang secukupnya  *I eat less than I think I should because I don’t have enough money for food* |  |  |  |
| 8. | Saya makan kurang daripada yang sepatutnya kerana tidak mempunyai wang yang cukup untuk makanan  *I eat less than I think I should because I don’t have enough money for food* |  |  |  |
| 9. | Saya tidak mampu memberikan makanan seimbang kepada anak-anak saya kerana saya tidak mampu  *I can’t afford to feed my child(ren) a balanced meal because I can’t afford that* |  |  |  |
| 10. | Saya tidak mampu memberikan makanan kepada anak-anak saya seperti yang saya patut  *I can’t afford to feed my child(ren) the way I think I should* |  |  |  |
| 11. | Anak-anak saya tidak makan secukupnya kerana saya tidak mampu membeli makanan yang mencukupi  *My child(ren) is/are not eating enough because I just can’t afford enough food* |  |  |  |
| 12. | Saya tahu anak-anak saya kadang kala lapar tetapi saya tidak mampu membeli lebih banyak makanan  *I know my child(ren) is/are hungry sometimes, but I just can’t afford more food* |  |  |  |
|  |  |  |  |  |
|  |  |  |  |  |
|  |  |  |  |  |
|  |  |  |  |  |
|  |  |  |  |  |
|  |  |  |  |  |

| \| J. REKOD DIET 24-JAM / *24-HOUR DIETARY RECALL* \| \| --- \|   Saiz Hidangan:  Sudu Teh =5 gm Sudu =15 gm Cawan = 120 gm  Mangkuk = 240 gm Gelas = 240 gm  Serving sizes:  Teaspoon =5 gm Table spoon =15 gm 1 soup cup = 120 gm  1 cup = 240 gm 1 glass = 240 gm  Hari 1 / *Day 1*: ____________________________ | | | |
| --- | --- | --- | --- | --- |
| SAJIAN  *MEAL TIME* | MAKANAN  *FOOD* | SAIZ HIDANGAN  *SERVING SIZES* | BERAT  *WEIGHT* |
| Sarapan  *Breakfast* |  |  |  |
| Minum Pagi  *Mid-morning snacks* |  |  |  |
| Makan Tengahari  *Lunch* |  |  |  |
| Minum Petang  *Tea-time* |  |  |  |
| Makan Malam  *Dinner* |  |  |  |
| Snek Malam  *Supper* |  |  |  |

| K. SOAL-SELIDIK KEKERAPAN MAKANAN / *SEMI-FOOD FREQUENCY QUESTIONNAIRE* | | | | | | |
| --- | --- | --- | --- | --- | --- | --- |
| Berapa kerap anda makan makanan-makanan di bawah?  *How often do you eat foods from each of the following categories?* | | | | | | |
| 1. BIJIRIN & PRODUK BERASASKAN BIJIRIN  *CEREAL & CEREAL-BASED PRODUCTS* | | Kali/hari  *Times/day* | Kali/minggu  *Times/week* | Kali/bulan  *Times/month* | Tak Pernah  *Never* | Saiz hidangan  *Serving size* |
| 1.1 | Nasi Putih  *White rice* |  |  |  |  |  |
| 1.2 | Nasi Putih (Beras Rebus)  *Parboiled rice* |  |  |  |  |  |
| 1.3 | Nasi perang / multibijirin / merah / liar  *Brown / multigrain / red / wild rice* |  |  |  |  |  |
| 1.4 | Bubur Nasi  *Rice porridge* |  |  |  |  |  |
| 1.5 | Nasi goreng  *Fried rice* |  |  |  |  |  |
| 1.6 | Nasi Lemak  *Nasi lemak* |  |  |  |  |  |
| 1.7 | Nasi ayam /nasi minyak / nasi biryani  *Chicken rice / oily rice / biryani rice* |  |  |  |  |  |
| 1.8 | Bihun goreng  *Fried meehoon* |  |  |  |  |  |
| 1.9 | Bihun / Laksa sup  *Meehoon / laksa soup* |  |  |  |  |  |
| 1.10 | Thosai  *Thosai* |  |  |  |  |  |
| 1.11 | Idli / Putu mayam  *Idli / putu mayam* |  |  |  |  |  |
| 1.12 | Mee goreng  *Fried noodles* |  |  |  |  |  |
| 1.13 | Mee sup  *Noodle soup* |  |  |  |  |  |
| 1.14 | Pasta  *Pasta* |  |  |  |  |  |
| 1.15 | Spageti  *Spaghetti* |  |  |  |  |  |
| 1.16 | Capati  *Capati* |  |  |  |  |  |
| 1.17 | Roti canai  *Roti canai* |  |  |  |  |  |
| 1.18 | Mee segera  *Instant noodles* |  |  |  |  |  |
| 1.19 | Bijirin sarapan sedia makan (eg. cornflakes)  *Ready-to-eat-cereals (eg. cornflakes)* |  |  |  |  |  |
| 1.20 | Oat/ muesli  *Oats/muesli* |  |  |  |  |  |
| 1.21 | Kentang  *Potaoes* |  |  |  |  |  |
| 1.22 | Ubi keledek  *Sweet potatoes* |  |  |  |  |  |
| 1.23 | Keladi  *Yam* |  |  |  |  |  |
|  | *Bijirin lain:*  *Other cereals:* |  |  |  |  |  |
| 2. SAYUR-SAYURAN  *VEGETABLES* | | Kali/hari  *Times/day* | Kali/minggu  *Times/week* | Kali/bulan  *Times/month* | Tak Pernah  *Never* | Saiz hidangan  *Serving size* |
| 2.1 | Sayuran hijau  *Green leafy vegetables* |  |  |  |  |  |
| 2.2 | Sayur buahan (eg. tomato, terung, labu)  *Fruits vegetables (eg. tomato, eggplant, pumpkin)* |  |  |  |  |  |
| 2.3 | Sayuran kobis (eg.kobis bunga, brokoli)  *Cruciferous (cauliflower, broccoli)* |  |  |  |  |  |
| 2.4 | Sayuran berkacang (eg. kacang panjang, kacang buncis)  *Leguminous vegetables (long beans, French beans)* |  |  |  |  |  |
| 2.5 | Akar/ubi (eg ubi bit, sengkuang, lobak merah)  *Roots (beetroot, sengkuang, carrot)* |  |  |  |  |  |
| 2.6 | Sayuran mentah (salad, ulam)  *Uncooked vegetables (salad, ulam)* |  |  |  |  |  |
|  | *Sayur-sayuran lain:*  *Other vegetables:* |  |  |  |  |  |
| 3. BUAH-BUAHAN  *FRUITS* | | Kali/hari  *Times/day* | Kali/minggu  *Times/week* | Kali/bulan  *Times/month* | Tak Pernah  *Never* | Saiz hidangan  *Serving size* |
| 3.1 | Buah buahan tempatan (eg. betik, pisang, tembikai)  *Local fruits (eg. papaya, banana, watermelon)* |  |  |  |  |  |
| 3.2 | Buah-buahan import (eg. epal, oren, kiwi, strawberi)  *Imported fruits (eg. apple, orange, kiwi, strawberry)* |  |  |  |  |  |
| 3.3 | Buah buahan kering (eg. kismis, kurma, prun)  *Dried fruits (eg. raisins, dates, prunes)* |  |  |  |  |  |
| 3.4 | Buah dalam tin  *Canned fruits in syrup* |  |  |  |  |  |
| 3.5 | Jus Buah-buahan segar  *Fresh fruit juices* |  |  |  |  |  |
|  | *Buah- buahan lain:*  *Other fruits:* |  |  |  |  |  |
| 4. DAGING & PRODUK DAGING, KEKACANG & BIJIAN  *FRUITS* | | Kali/hari  *Times/day* | Kali/minggu  *Times/week* | Kali/bulan  *Times/month* | Tak Pernah  *Never* | Saiz hidangan  *Serving size* |
| 4.1 | Daging merah (lembu, kambing, babi)  *Red meat (beef, mutton, pork)* |  |  |  |  |  |
| 4.2 | Daging putih (ayam, itik, arnab)  *White meat (chicken, duck, rabbit)* |  |  |  |  |  |
| 4.3 | Ikan  *Fish* |  |  |  |  |  |
| 4.4 | Makanan laut (udang, kerang)  *Seafood (prawns, clams)* |  |  |  |  |  |
| 4.5 | Ikan masin kering / makanan laut (termasuk ikan bilis)  *Dried salted fish / seafood (including anchovies)* |  |  |  |  |  |
| 4.6 | Telur (ayam, itik, puyuh)  *Eggs (hen, duck, quill)* |  |  |  |  |  |
| 4.7 | Sosej/nugets  *Sausages / nuggets* |  |  |  |  |  |
| 4.8 | Kepingan bakon / ham  *Bacon / ham / slices* |  |  |  |  |  |
| 4.9 | Susu segar  *Fresh milk* |  |  |  |  |  |
| 4.10 | Susu tepung /UHT penuh krim  *Milk, full cream, powdered/UHT* |  |  |  |  |  |
| 4.11 | Susu tepung /UHT skim  *Milk, skim/low fat, powdered/UHT* |  |  |  |  |  |
| 4.12 | Susu pekat manis  *Condensed milk* |  |  |  |  |  |
| 4.13 | Krim bukan susu  *Non-dairy creamer* |  |  |  |  |  |
| 4.14 | Keju  *Cheese* |  |  |  |  |  |
| 4.15 | Yogurt / tairu / mooru / dadih  *Yogurt / tairu / mooru / dadih* |  |  |  |  |  |
| 4.16 | Susu soya  *Soy milk* |  |  |  |  |  |
| 4.17 | Tofu / tofu Jepun / tempeh  *Tofu / Japanese tofu / tempeh* |  |  |  |  |  |
| 4.18 | Kacang (eg kacang tanah, almond, pistachio, badam)  *Nuts (eg peanuts, almonds, pistachios, walnuts)* |  |  |  |  |  |
| 4.19 | Bijian (kuaci)  *Seeds (kuaci)* |  |  |  |  |  |
|  | *Makanan berasaskan protein lain:* |  |  |  |  |  |
| 5. LEMAK & MINYAK  *FATS & OILS* | | Kali/hari  *Times/day* | Kali/minggu  *Times/week* | Kali/bulan  *Times/month* | Tak Pernah  *Never* | Saiz hidangan  *Serving size* |
| 5.1 | Minyak kelapa sawit  *Palm oil* |  |  |  |  |  |
| 5.2 | Minyak bunga matahari / zaitun  *Sunflower / olive oil* |  |  |  |  |  |
| 5.3 | Minyak campuran  *Blended oil* |  |  |  |  |  |
| 5.4 | Jenis lain minyak sayur-sayuran:  *Other type of vegetable oil:*  _______________________________ |  |  |  |  |  |
| 5.5 | Minyak sapi  *Ghee* |  |  |  |  |  |
| 5.6 | Vanaspati  *Vanaspati* |  |  |  |  |  |
| 5.7 | Marjerin  *Margerine* |  |  |  |  |  |
| 5.8 | Lemak babi / haiwan  *Lard / animal fat* |  |  |  |  |  |
|  | *Lemak dan minyak yang lain:*  *Other type of fats & oils* |  |  |  |  |  |
| 6. MINUMAN  *BEVERAGES & DRINKS* | | Kali/hari  *Times/day* | Kali/minggu  *Times/week* | Kali/bulan  *Times/month* | Tak Pernah  *Never* | Saiz hidangan  *Serving size* |
| 6.1 | Kopi  *Coffee* |  |  |  |  |  |
| 6.2 | Teh Tarik  *Teh Tarik* |  |  |  |  |  |
| 6.3 | Teh (eg. O, Cina, hijau)  *Tea (eg. Black, Chinese, Green)* |  |  |  |  |  |
| 6.4 | Minuman bertenaga (eg. Milo, Horlicks)  *Malt drink (eg. Milo, Horlicks)* |  |  |  |  |  |
| 6.5 | Minuman beralkohol – wain  *Alcoholic drink – wine* |  |  |  |  |  |
| 6.6 | Minuman beralkohol – bir  *Alcoholic drink – beer* |  |  |  |  |  |
| 6.7 | Minuman beralkohol – likur  *Alcoholic drink - liquor* |  |  |  |  |  |
| 6.8 | Kordial  *Cordials* |  |  |  |  |  |
| 6.9 | Minuman berkarbonat  *Carbonated / soft drinks* |  |  |  |  |  |
|  | *Minuman lain:*  *Other beverages / drinks:* |  |  |  |  |  |
| 7. LAIN-LAIN  *MISCELLANEOUS* | | Kali/hari  *Times/day* | Kali/minggu  *Times/week* | Kali/bulan  *Times/month* | Tak Pernah  *Never* | Saiz hidangan  *Serving size* |
| 7.1 | Makanan segera  *Fast foods* |  |  |  |  |  |
| 7.2 | Coklat  *Chocolates* |  |  |  |  |  |
| 7.3 | Biskut  *Cookies / biscuits* |  |  |  |  |  |
| 7.4 | Kuih tempatan  *Local kuih* |  |  |  |  |  |
| 7.5 | Kek  *Cake* |  |  |  |  |  |
| 7.6 | Ais krim  *Ice cream* |  |  |  |  |  |
| 7.7 | Gula  *Sugar* |  |  |  |  |  |
| 7.8 | Madu  *Honey* |  |  |  |  |  |
|  | *Lain-lain:*  *Others:* |  |  |  |  |  |

8. SUPLEMEN

*SUPPLEMENTS*

| Jenis  Type | Kekerapan  *Frequency* | | | |
| --- | --- | --- | --- | --- |
|  | Setiap hari  *Every day* | >sekali / seminggu  *> Once / week* | Sekali seminggu  *Once a week* | 1 – 3 kali / sebulan  *1 – 3 times/month* |
| 8.1 Multivitamin  *Multivitamin* |  |  |  |  |
| 8.2 Vitamin B kompleks  *B Complex* |  |  |  |  |
| 8.3 Vitamin C  *Vitamin C* |  |  |  |  |
| 8.4 Kalsium  *Calcium* |  |  |  |  |
| 8.5 Zat besi  *Iron* |  |  |  |  |
| 8.6 Minyak ikan  *Fish oil* |  |  |  |  |
| 8.7 Minyak evening primrose  *Evening primrose oil* |  |  |  |  |
| 8.8 Herba Cina / ginseng  *Chinese herbs/ginseng* |  |  |  |  |
| 8.9 Serbuk protein / whey  *Protein powder / whey* |  |  |  |  |
| *Others:* |  |  |  |  |

| L. MAKLUMAT PERUBATAN / *MEDICAL INFO* | | | | | | | | | | | | |  |  |
| --- | --- | --- | --- | --- | --- | --- | --- | --- | --- | --- | --- | --- | --- | --- |
|  |  | | |  | | |  | | | |  | |  |  |
| 1. | Adakah anda pernah mengalami masalah kesihatan yang berikut?  *Presence of the following:* | | | | *(1) Jaundice*  *(2) Pallor*  *(3) Clubbing* | | | | *(4) Koilonychias*  *(5)Lymphadenopathy*  *(6) Oedema* | | | |  |  |
| 2. | Adakah anda mengambil sebarang ubat-ubatan sekarang?  *Are you taking any medications for your disease?* | | | | (1) Ya / *Yes* | | | | | (2) Tidak */ No*  *(sila ke S4 / skip to Q4)* | | |  |  |
|  |  | | | |  | | | | |  | |  |  |  |
| 3. | Apakah ubat-ubatan yang anda ambil?  *What are those medications?* | Kencing manis  *Diabetes mellitus* | Darah tinggi  *Hypertension* | | | Kolesterol tinggi  *Hypercholesterolemia* | | | | Lain-lain  *Others* | | |  |  |
|  |  |  |  | | |  | | | |  | | |  |  |
|  |  | | | |  | | | | |  | |  |  |  |
| 4. | Adakah mana-mana ahli keluarga anda (ibubapa, adik beradik, anak-anak) mempunyai sebarang sejarah penyakit?  *Has/had any of your family members (parents, siblings, children) a history of any disease?* | | | | | | | (1) Ya / *Yes* | (2) Tidak / *No* | | | | |  |
|  |  | | | | | | |  | |  | |  | | |
| 5. | Apakah penyakit-peyakit yang mereka ada/pernah ada?  *Which of the listed diseases your family members has/had?* | | | | | | | (1) Kencing manis / *diabetes mellitus*  (2) Darah tinggi / h*ypertension*  (3) Kolesterol tinggi / *hypercholesterolemia*  (4) Lain-lain / *others:*  __________________________ | | | | | |  |

| *Perempuan sahaja / Female participants only* | | | | |
| --- | --- | --- | --- | --- |
|  |  |  |  |  |
| 6. | Pernah ada kencing manis ketika mengandung?  *Any history of diabetes reported during pregnancy?* | (1) Ya / *Yes* | (2) Tidak /*No* | (3) Tidak berkaitan/*NA* |
|  |  |  |  |  |
| 7. | Pernah ada darah tinggi ketika mengandung?  *Any history of hypertension reported during pregnancy?* | (1) Ya / *Yes* | (2) Tidak /*No* | (3) Tidak berkaitan/*NA* |

| PENYAKIT ARTERI KORONARI / *CORONARY ARTERY DISEASE* | | | |
| --- | --- | --- | --- |
| 8. | Pernahkah doktor memberitahu anda aliran darah ke jantung anda terhalang, juga dikenali sebagai penyakit arteri koronari? Halangan sedemikian boleh menyebabkan sakit dada yang juga dikenali sebagai angina.  *Has a doctor ever told you that you have a blockage in the blood flow to your heart, also called CAD? Such blockage can lead to chest pain, also called angina?* | (1) Ya / *Yes* | (2) Tidak / *No* |
|  |  |  |  |
| 9. | Pernahkah anda mendapat sakit atau tekanan dada dalam 6 bulan yang lepas?  *In the past 6 months have you had chest pain or pressure?* | (1) Ya / *Yes* | (2) Tidak /*No* |
|  |  |  |  |
| 10. | Adakah sakit atau tekanan tersebut disebabkan aktiviti fizikal atau stres?  *Was the chest pain or pressure brought on by physical activity or stress?* | (1) Ya / *Yes* | (2) Tidak / *No* |
|  |  |  |  |
| 11. | Adakah sakit atau tekanan dada tersebut pulih selepas berehat atau guna nitrogliserin?  *Was the chest pain or pressure relieved by rest or nitroglycerine?* | (1) Ya / *Yes* | (2) Tidak / *No* |
|  |  |  |  |
| 12. | Pernahkah doktor memberitahu anda telah diserang sakit jantung?  *Has a doctor ever told you that you had a heart attack?* | (1) Ya / *Yes* | (2) Tidak / *No* |

|  |  |  |  |
| --- | --- | --- | --- |
| PENYAKIT CEREBROVASKULAR / *CEREBROVASCULAR DISEASE* | | | |
| 13. | Pernahkah doktor memberitahu anda mendapat strok?  *Have you ever been told by a doctor that you have had a stroke?* | (1) Ya / *Yes* | (2) Tidak / *No* |
| 14. | Pernahkah doktor memberitahu anda mendapat TIA? Ia just dipanggil “*Transient Ischemic Attack*” atau strok amaran.  *Has a doctor ever told you that you have had a TIA? This is also called “Transient Ischemic Attack” or “warning stroke.”* | (1) Ya / *Yes* | (2) Tidak / *No* |
| 15. | Pernahkah anda mendapat simptom-simptom seperti strok, contohnya rasa lemah sebelah badan anda, gagap, sebelah mulut kendur, meleleh air liur atau kesukaran melihat, tetapi kembali normal dalam masa sehari?  *Have you ever developed sudden, stroke-like symptoms, for example, weakness on one side of your body, difficulty speaking, drooping of one side of your mouth, drooling, or trouble seeing, which completely returned to normal within a day?* | (1) Ya / *Yes* | (2) Tidak / *No* |
| PENYAKIT VASKULAR PERIFERAL / *PERIPHERAL VASCULAR DISEASE* | | | |
| 16. | Pernahkah doktor memberitahu anda mempunyai saluran darah, arteri-arteri ke kaki tersumbat? Ia juga dipanggil penyakit vaskular periferal.  *Has a doctor ever told you that you have blockages in the blood vessels, arteries to your legs, also called peripheral vascular disease?* | (1) Ya / *Yes* | (2) Tidak / *No* |
| 17. | Pernahkah anda mendapat kekejangan kaki atau kesakitan pada bahagian betis yang pulih selepas berehat dalam 6 bulan yang lepas? | (1) Ya / *Yes* | (2) Tidak / *No* |
|  | *During the past 6 months, have you had leg cramps or pain in your calf while walking, which was relieved by rest?* |  |  |
| NEUROPATI / *NEUROPATHY* | | | |
| 18. | Pernahkah anda rasa kehilangan sensasi atau rasa kebas di kaki dalam 6 bulan yang lepas?  *During the past 6 months, have you had no feeling or numbness in your feet?* | (1) Ya / *Yes* | (2) Tidak / *No* |
| 19. | Berapa kerap anda mempunyai kehilangan kawalan membuang air besar atau cirit-birit semasa tidur dalam 4 minggu yang lepas?  *During the past 4 weeks, how often have you had loss of bowel control or diarrhoea while sleeping?* | (1) Tidak pernah / *Never*  (2) 1 or 2 kali sebulan / *1-2 times a month*  (3) Sekali seminggu / *once a week*  (4) 2 or 3 kali seminggu / *2-3 times a week*  (5) Selalu / *Always* | |
| MASALAH BERKAITAN KAKI / *FOOT PROBLEMS* | | | |
| 20. | Pernahkah anda mendapat ulser di jari kaki, kaki atau bahagian bawah kaki dalam 6 bulan lepas?  *During the past 6 months, have you had ulcers on your toes, feet, or lower legs?* | (1) Ya / *Yes* | (2) Tidak / *No* |
| 21. | Pernahkah anda mendapat gangren pada jari-jari kaki?  *Have you ever had gangrene on any of your toes?* | (1) Ya / *Yes* | (2) Tidak / *No* |
| 22. | Pernahkah jari kaki atau kaki anda dipotong disebabkan kencing manis?  *Have you ever had any part of your toes or feet amputated because of diabetes?* | (1) Ya / *Yes* | (2) Tidak / *No* |
| MASALAH BERKAITAN MATA / *EYE PROBLEMS* | | | |
| 23. | Adakah anda mempunyai katarak?  *Do you now have cataracts?* | (1) Ya / *Yes* | (2) Tidak / *No* |
| 24. | Pernahkah doktor memberitahu anda mempunyai penyakit mata kencing manis atau retinopati?  *Has a doctor ever told you that you have retinopathy or diabetic eye disease?* | (1) Ya / *Yes* | (2) Tidak / *No* |
