# Supplementary material for: Relationship of sociodemographic and lifestyle factors and diet habits with metabolic syndrome (MetS) among three ethnic groups of the Malaysian population
Source: PLoS One. 2020 Mar 19;15(3):e0224054. doi: 10.1371/journal.pone.0224054 (PMC7082049; doi:10.1371/journal.pone.0224054)
Supplement: Attachment — Submitted filename: Answers to comments-3 PLoS One.docx [file pone.0224054.s003.docx]

**Answers to comments**

We thank the editor and reviewers for their kind feedback on improving the manuscript. Our reply to the concerns mentioned are as below.

| **Comments by editor / reviewer** | **Answer** | **Modifications in the manuscript** |
| --- | --- | --- |
| 1. Although sample size calculation was included, the issue on small sample size of this study remains a question. Authors could justify by showing the prevalence of MetS for each ethnicities, and the calculation of power of studies. | Prevalence of MetS across ethnicities have been added in the Results section.  While calculating the sample size at the start of the study, stratification was not the objective of the study.  However, post study calculation for Power has been performed for the 3 major ethnic groups in Johor revealing that except for the Malays, the study power for the Indians and the Chinese was pretty good (84.8% and 71.7%, respectively). In the previous local research on MetS in Johor, these minor ethnic groups had been underrepresented, however, in this study we ensured adequate representation of these minor ethnicities, so that associations could be seen also among all 3 major races residing in Johor. We have indicated this in the discussion section of the revised manuscript. | Please see the highlighted lines (line 8-10) of the last paragraph on page 25 of the revised manuscript. |
| 2. There is a major concern regarding the sample size and the interpretation of data in the paper. The total number of samples is 481 and when it was stratified into 3 ethnic groups, Malay, Chinese and Indian have the number of 147, 228 and 106, respectively. These sample sizes are too small and cannot be settled with valid and reliable results, especially for Table 3. The problem of small sample size is simply reflected on the wide 95% CI in Table 3 where the authors claimed that the higher age groups of Chinese and Indian were associated with MetS, low physical activity in Chinese and fast finishing meals in Malay were in favour of greater risk of MetS. As the 95% CI is extremely wide, it indicates the very weak association of variables with MetS despite the p value is less than 0.05. In addition, the method in calculating the sample size was not written in detail and also it is not clear how the estimated sample size was calculated for each ethnic group. | Most respectfully, we would beg to disagree with the reviewer on this point. The results showed in table 3 needs to be seen in the continuation of table 2. Table 2 shows the effect of lifestyle and diet factors with MetS overall. For example, overall, increasing age increases the odds of MetS (a significant finding with precise confidence intervals). This characteristic was then further viewed within each ethnicity, yielding results that corroborated with the findings in table 2.  Stratification was never intended for this study The objective was to see which ethnicity or ethnicities influence the outcome in presence of the risk factor, as seen in the overall target sample. However, we do agree that stratification does lower the study power and widens the confidence intervals indicating weak associations (as shown among Indians with age in our study). Despite the weaker association, the study does indicate that an association exists. Other researchers have also shown these findings in research conducted in similar settings.  We have modified our sentences in the discussion section to highlight this aspect and indicated that for future research on such populations stratification by ethnicity is likely to yield more conclusive results. | Please see the highlighted lines (line 6-12) of the last paragraph of the Discussion section on page 26 in the revised manuscript. |
| 3. A few relevant variables are missing in the analysis. Among them, wealth/income index is the most relevant variable for the sociodemographic characteristics but it is not included in the analysis. | We agree that the wealth index is important to explore the sociodemographic characteristics. However, this is a composite variable; requiring detailed information on the households’ assets, house construction, water access, etc, which was beyond the scope of this research.  We had a question in our questionnaire to inquire the monthly incomes. However, this being a sensitive question, more than half of the recruited participants refused to furnish this information. Consequently, this variable could not be included in the final analysis.  We have indicated in the discussion section that the association of wealth index with MetS needs to be explored in future research. | Please see lines 4-6 of the last paragraph of the Discussion section on page 26 of the revised manuscript. |
| 4. The title of paper does not reflect the actual population that was studied. I would suggest to replace “a multi-ethnic Asian population” to “three ethnic groups of the Malaysian population” in the title. | We greatly appreciate this useful suggestion and have made this modification, and the title has been changed as suggested. | Please see the highlighted title on page 1 of the revised manuscript. |
| 5. The study was taken place in Johor, however, no explanation of why Johor is selected in this study. Can the population in Johor be the representative of the nation? | Johor represents the other states in Malaysia in terms of ethnic distribution, based on statistics by the Department of Statistics, Malaysia.  As we employed a non-random sampling technique to select study subjects, we cannot say that the study population is representative of Malaysia. However, the study is representative of similar settings in Malaysia. We have added the above mentioned lines in the methods section. | Please see the highlighted lines in the Methods section on page 8. |
| 6. Page 15, line 3 from the bottom of paragraph: “….41.6% at the age group of 40-49 years…..” Instead of 41.6%, it was 41.8% written in Table 1. | This oversight has been corrected. | Please see the highlighted percentage on page 15 of the Results section |
| 7. Page 24, line 4 from the top of paragraph: “Our results show the prevalence of MetS and its components to be comparatively lower among Chinese than in the Malays and the Indians, and this could be attributed to better lifestyle choices, including physical activity”. The sentence is overstated since there is not enough evidence to show Chinese have better lifestyle choices except the physical activity. | We agree with the reviewer’s comment and have revised the statement in this regard and have removed the words “lifestyle choices”. | Please see the highlighted lines (lines 4-6) on page 24 of the revised manuscript. |
| 8. The following references are similar studies and shall be mentioned in the paper: - Kaur et al. (2018) Journal of Immigrant and Minority Health 20, 1380-1386. - Narayanan et al. (2011) Metabolic Syndrome and Related Disorders 9, 389-395. - Tan et al. (2011) Metabolic Syndrome and Related Disorders 9, 441-451. | These references have been incorporated in the manuscript. | Please see reference number 29 (page 35), 40 and 43 (on page 37) in the reference section of the revised manuscript. |
| 9. Some defining criteria of metabolic syndrome (page 12) are inaccurately written - the definitions of raised serum triglycerides and low HDL are missing the symbols of "equals to or more than"and "less than" respectively. | Thanks. These have been rectified. | Please see the highlighted lines on page 12 of the revised manuscript. |
| 10. Isolated grammatical errors are present including those listed below: 1)page 23; paragraph 2; line 1 2)page 24; paragraph 1; line 10 2) page 25; paragraph 2; line 6-7 | Thanks.These have been rectified. | Please see the highlighted lines on page 23-25 of the revised manuscript. |
